# History of Bone Grafts in Spine Surgery

**DOI:** 10.7759/cureus.24655

**Published:** 2022-05-01

**Authors:** Gilad A Hampel, Emre Yilmaz, Chrissie Massrey, William Clifton, Joe Iwanaga, Marios Loukas, R. Shane Tubbs

**Affiliations:** 1 Department of Structural & Cellular Biology, Tulane University School of Medicine, New Orleans, USA; 2 Department of Trauma Surgery, Ruhr University, Bochum, DEU; 3 Department of Anatomical Sciences, St. George's University, St. George, GRD; 4 Department of Orthopedic Surgery, Columbia Medical Center, New York, USA; 5 Department of Neurosurgery, Tulane University School of Medicine, New Orleans, USA; 6 Department of Neurosurgery and Ochsner Neuroscience Institute, Ochsner Health System, New Orleans, USA

**Keywords:** surgery, bone graft substitutes, spine, spinal fusion, history, bone graft

## Abstract

Bone grafting replaces damaged or missing bone with new bone and is used for surgical arthrodesis. Patients benefit from a huge variety of bone graft techniques and options for spinal fusions. This article reviews the rich history of bone grafts in surgery with particular emphasis on spinal fusion. During the early years of bone grafting in spine surgery, bone grafts were used on tuberculosis patients, and the structural support of the graft was most the important consideration. Between 1960 and 2000, many advances were made, specifically in the use of bone graft substitutes. The field of bone grafts in spine surgery has evolved rapidly since first described.

## Introduction and background

Bone grafts are widely used in orthopedic surgery, with materials progressively evolving over time from gold plates to advanced molecular approaches harnessing bone morphogenetic proteins (BMP) and stem cells. Bone grafting dates from the Neolithic age, when a Peruvian tribal chief‘s frontal bone was repaired with a gold plate [1]. There is some evidence that the Aztecs used wooden sticks to repair bone fractures [1]. Furthermore, two skulls in Ishtkunui were found by Jagharian, an anthropologist who noticed elements of bone grafts within skulls dating back to around 2000 BCE. The first skull he found showed a piece of animal bone that had been inserted into an injured area of a skull, demonstrating very early attempts at xenografting [2]. The first reports of bone grafts in spine surgery in the 20th century began with Hibbs and Albee, who used bone chips from the tibia and transverse processes [3-4]. Such grafts have evolved over the years. More recent examples of bone graft materials include bioactive glass and biologicals such as bone matrix protein. Therefore, this aspect of spine surgery is continuously evolving. Such materials can be osteoinductive, osteogenic, or osteoconductive (Table 1).

**Table 1:** Various bone substitutes and their ability to act as osteoconductive, osteogenic, or osteoinductive BMP: bone morphogenetic protein

Osteoconductive	Osteogenic	Osteoinductive
Hydroxyapatite	BMP	Calcium phosphate
Coralline hydroxyapatite		Calcium sulfate
Collagraft		
Autograft	Autograft	Autograft
Allograft		Allograft

In view of the rich history of bone grafts, the purpose of this paper is to highlight the evolution of bone grafts in relation to spine fusion in more detail.

## Review

Ancient history

There is evidence of orthopedic surgeries performed in Ancient Egypt (656-535 BCE). Many Egyptians examined after death showed evidence of limb prostheses, as illustrated by the “black leg miracle,” a medieval painting depicting a homoplastic transplant of an Ethiopian limb onto the sacristan Justinian [5]. More modern techniques were developed beginning in the 1600s by Job van Meekeren. Meekeren is credited with the first heterologous bone graft procedure in 1668, when he grafted a dog skull fragment into the skull of an injured soldier. When the soldier wanted the graft removed for religious reasons, Meekeren observed the bony fusion. When he looked to see if he could remove the fragment, he noticed it had been fully incorporated into the soldier’s skull [6].

Modern history

Sub-periosteal resection was the standard treatment of non-union fusions in the nineteenth century until 1820, when Philips von Walter performed the first autologous bone graft on a skull in Germany [7]. In 1861, Leopold Ollier published “Traite de la regeneration des os,” which was the first paper to define the term bone graft. Like Meekeren, Ollier also noticed the importance of the periosteum for osteogenesis [8]. Barth, however, described the importance for bone regeneration of not only the periosteum but also the bone itself and the marrow. The first homologous bone graft was not performed until 1880; William Macewen used the tibia of a child with rickets to treat a child burdened with an infection in his humerus [6]. Phelps (1891) was the first to recognize the importance of vascularization for the success of bone grafting when he took a piece of bone from a dog and transplanted it into a boy’s tibia. He kept the dog and the boy attached to each other for two weeks so the blood could circulate between them. This allowed new bone to grow in the boy [9]. 

Twentieth-century spinal fusion had two brilliant minds at the helm, Russell Hibbs and Fred Albee. These two individuals paved the way for advanced techniques in orthopedic surgery still used today. Originally, spinal fusions were developed to remediate diseased tissue in tuberculosis and to correct mild to extreme spinal deformations from scoliosis. Hibbs and Albee both used bone grafts to achieve union; Hibbs used fragments of the spinous processes and laminae and Albee used grafts from the tibia [10]. Hibbs’s interest in the pathology of tuberculosis ultimately led him to find a way to immobilize the diseased tissue of a vertebral column completely once fused, and thus “make development of deformity impossible” [10]. On January 9, 1911, Hibbs performed the first spinal fusion on a boy with spinal tuberculosis. His ingenuity was evident, as he also implemented grafts of the laminae and lateral articulations [10]. He admitted that in very young individuals, grafting from the tibia seemed to be better for preserving aspects of the laminae and lateral spine, presumably because those spines had yet to achieve significant development.

**Figure 1:**
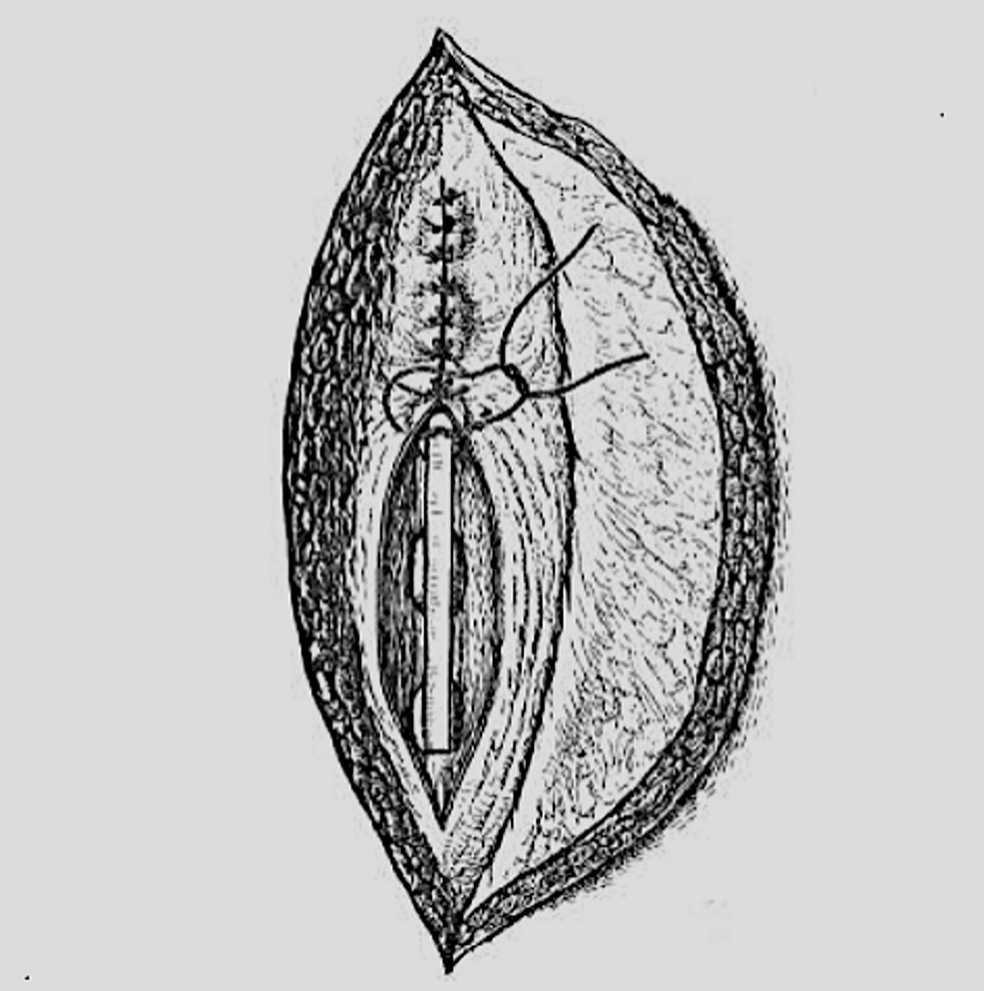
A drawing that shows insertion of bone graft into five thoracic spinous processes with soft tissue suturing (After Albee, 1915)

In the same year, Albee successfully harvested tibial bone grafts, incorporating them into the spinous processes of his patients (Figure 1).

A detailed molecular explanation of why autogenous and homogenous grafting material was optimal had not yet been elucidated, but Albee recognized that grafts “derived from the same individual were most trustworthy” [4]. In one case, Albee achieved approximation between the inferior aspects of the superior lamina and the superior aspect of the inferior lamina with a “heavy kangaroo tendon,” which progressed to a successful union after chips of the spinous processes were placed across this interface. This was seen at the time as ingenious, interesting, and welcome [10]. Albee suggested that grafting fruit trees was analogous to bone tissue grafting; just as the bark, sap, and wood are firmly approximated to the recipient branch, so there must be an intimate juxtaposition between the graft and recipient bone across the area of contact. Albee noted that the “technique is not difficult because it has to do with plane surfaces” [4]. His thorough understanding of Wolff’s law, asserting it to be paramount in achieving successful restoration of the bone’s original tensile strength, was apparent in his remark: “the external shape of the bone is the result of functional adaptation, [allowing] bones or grafts in their altered positions and relationships to meet the new and abnormally directed stress thrown upon them” [4].

By 1915, the procedures of Hibbs and Albee had been performed in hundreds of cases, including Farrell’s osteoplastic treatments of Pott’s disease [3-4]. In 1920, Sheen described a case of a 22-year-old girl with a tuberculous spine. He grafted a six-inch-long tibial graft into her back. The six-month radiographical follow-up showed that “the graft is visible as a long, curved rod rounded at the ends.” The author concluded that “there can be no doubt that the graft has maintained its vitality” [11].

**Figure 2:**
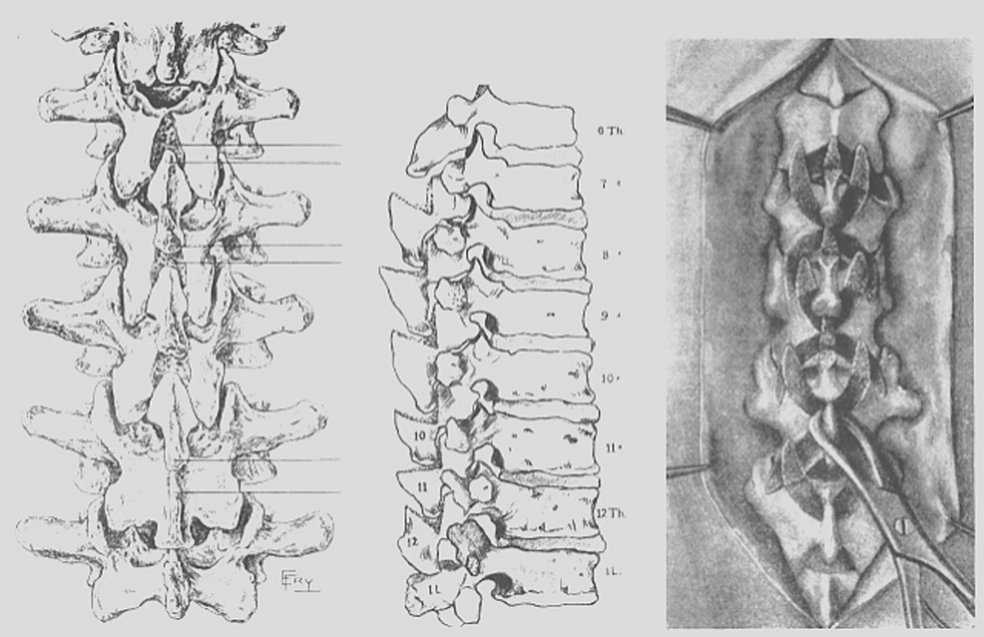
Illustration showing the first spinal fusion operation performed The illustration to the far right shows the use of the pieces from the lamina and the scrapings of the lateral articulations.

**Figure 3:**
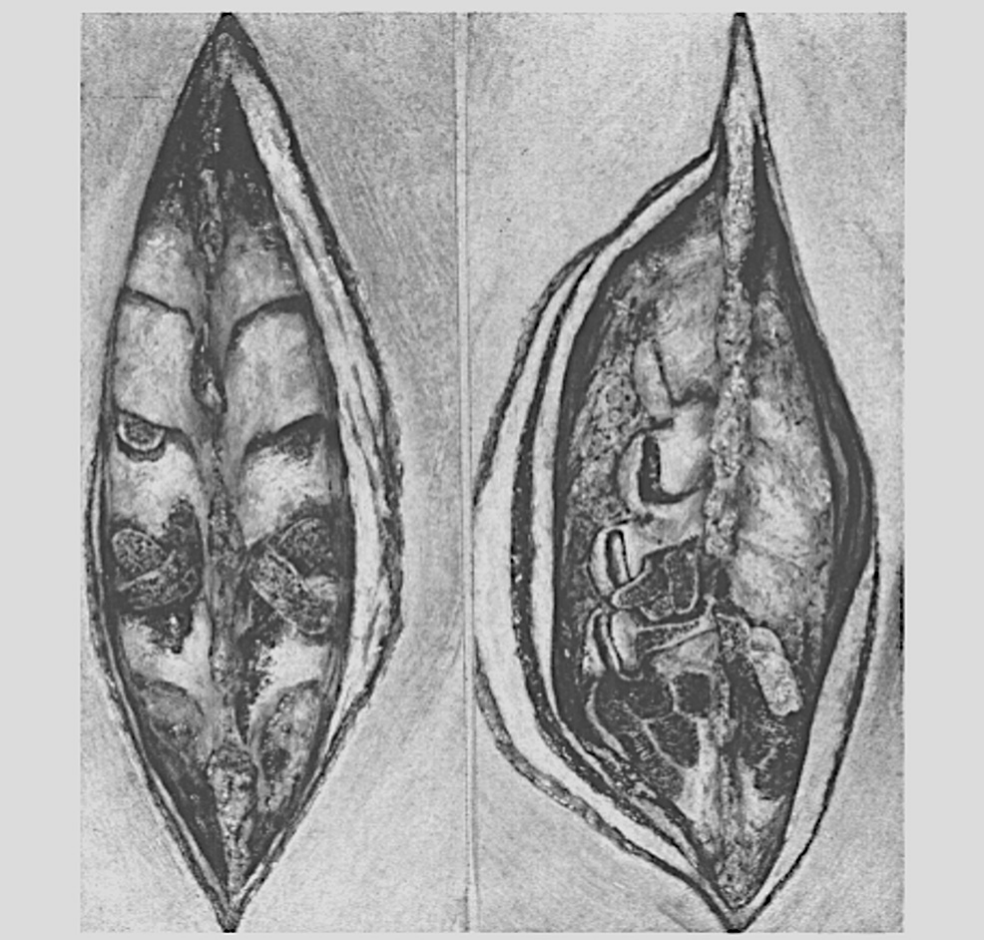
Stages of a spinal fusion with the cutting of the spinous processes and ligamentum flavum, excision of the posterior articular capsules, articular cartilage, and insertion of bone chips (left) The same procedure in the lumbar spine is shown to the right. (After Dommisse, 1959)

In 1921, Radulesco modified Albee’s tibial graft procedure by using half of a rib with the periosteum intact [12]. Other materials used for bone grafts included bovine bone by Brown and Kleinberg [13-14]. Additional indications for spinal fusions with grafting were generated, and in 1914 and 1937, the first fusions were performed to correct mechanical conditions (e.g., hemivertebra) and disc herniation, respectively (Figure 2 and Figure 3) [4,10].

**Figure 4:**
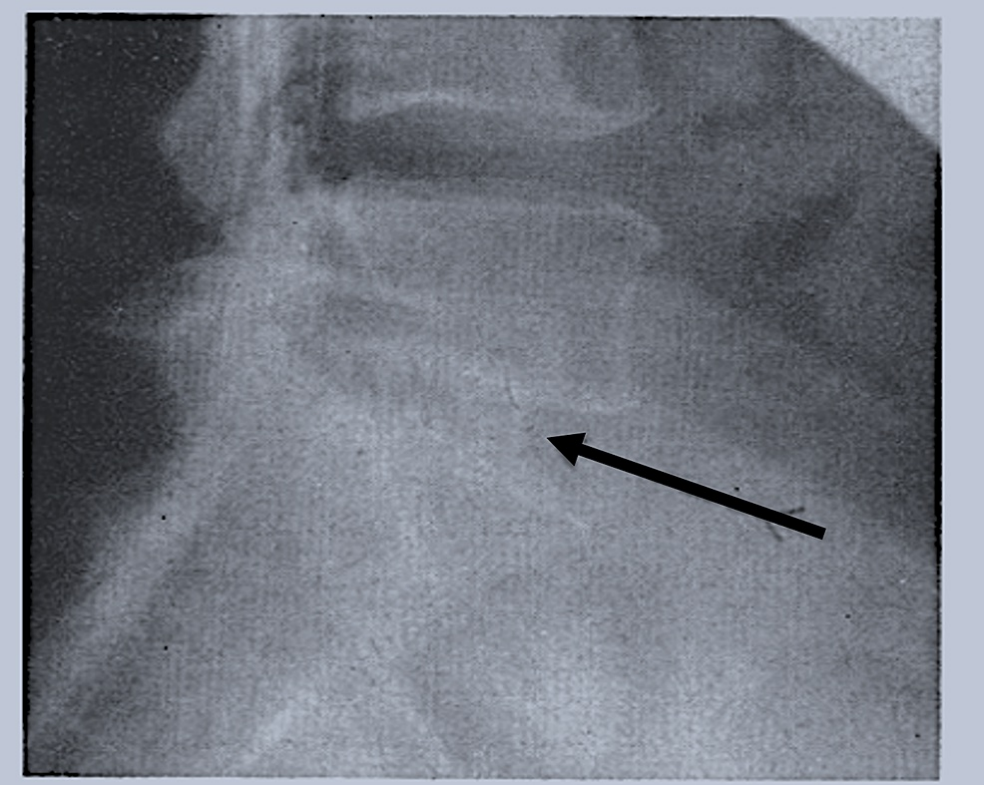
Lateral view of the lumbosacral spine after interbody spinal fusion (arrow) using calvaria (After Cloward, 1958)

Petter also used a splintered rib to produce firmer fixation following the destruction of the articular facets, resulting in a larger surface area of graft covering the entire surface of the laminae [15]. The middle of the 20th century saw another advance in autologous and homologous graft procedures: Abbott modified the Cloward interbody fusion by using autologous and homologous grafts from harvested discs of cranial bones (Figure 4) [16].

The anterior approach to the cervical spine was first introduced in the early 1950s [17-19]. In 1952, Bailey and Badgely used an autologous on-lay strut bone graft for anterior decompression and fusion in a patient with a cervical lytic lesion [17].

**Figure 5:**
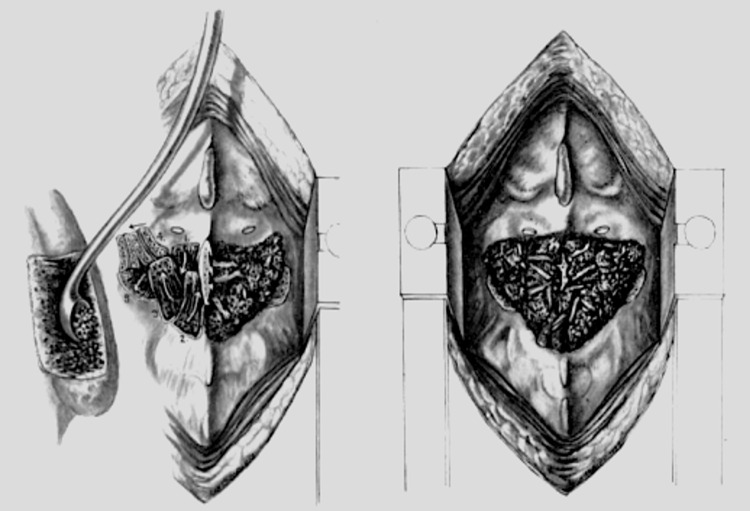
The process of using cancellous bone from the posterior superior iliac spine for spinal fusion (left). The completed spinal fusion (right). (After Dommisse, 1959)

In 1955, Robinson and Smith reported the first use of a tri-cortical horseshoe-shaped iliac crest graft for anterior cervical discectomy and fusion for spondylosis [19]. In 1959, Boucher mentioned work with the posterior superior iliac spine (PSIS), where the external cortex of the iliac spine was removed, and the inner spongy bone was removed with a curette and replaced in the fusion cavity [20]. Dommisse (1959) also claimed that lumbosacral spine fusions were successful only if both sides of the PSIS were grafted (Figure 5).

He noted that shear strain was a limiting factor that had kept non-union rates high in the past; ensuring maximal contact between graft and graft bed was a crucial detail in establishing fusion [21].

There were many advances in bone grafts between 1960 and 2000, specifically in the use of bone graft substitutes. In 1965, Urist discovered bone morphogenic proteins (BMPs) [21-22], which led to further improvement of growth factor-enhanced absorbable collagen sponges [23]. During the 1970s, porous metals were considered possible bone graft substitutes due to their physical strength [24]. The first use of ceramics for spinal bone grafts was reported in 1979 by Shima et al., where 20 dogs underwent an anterior cervical discectomy and interbody fusion. The first use of ceramics in a human spine was reported in 1991. Hase et al. described the use of ceramic laminae in bilateral open laminoplasty for cervical myelopathy [25]. During the 1990s, the use of ceramics in spine surgery was reported by Ransford et al. in a study that compared ICBG with β-TCP (tricalcium phosphate) [26].

Lindholm et al. analyzed the use of demineralized bone grafts for inducing new bone formation during the 1980s. One of the first reports described the capability of grafts consisting of demineralized bone matrix (DBM) combined with autogenous bone marrow to enhance fusion in the thoracic and lumbar spines of rabbits [27-28]. Since then, DBM has been widely used as a potential bone graft enhancer, extender, or substitute. In 1988, stainless steel interbody cages were introduced [29]. Since then, interbody cage technology has improved to now use polyetherketone, titanium, and carbon fiber-reinforced polymers [30]. Curylo reported the first case of autologous bone marrow aspirate used for posterolateral fusion in a rabbit model in 1999 [31]. Finally, during the early 2000s, tantalum became the first porous metal to be used as an implant in bone grafting [32-33].

Pitfalls of bone grafts

Iliac crest harvest sites are used because they have been shown to have increased expression of natural BMPs, BMP receptors, and other factors important for graft success [34], making them osteogenic, osteoinductive, and osteoconductive. While grafting from the iliac crest has been shown to have many benefits, it also has its pitfalls. Since autografts come from the same individual, they may have complications, including chronic pain from the graft site, decreased sensation in the graft site, infection, and the possibility of hematoma development at the harvest site [35-36]. Additionally, iliac crest bone grafts have been shown to increase donor site morbidity (up to approximately 50%, although this decreases over time) in that they can lead to both an increase in operating time and increased length of stay in the hospital [37-38] as compared to grafts from other body sites. Due to these complications, much research has been done to counteract how much of graft is needed for a successful surgery. Synthetic compounds called extenders are often used as additives to auto/allografts and have been helpful in limiting the amount of graft that needs to be taken from the iliac crest [39-41].

An alternative to the autograft is an allograft in which bone is taken from another person. Allografts are osteoconductive and osteoinductive, however, they lack osteogenic properties, as those cells are lost when the allograft is sterilized. A popular form of allograft in spine surgery is demineralized bone matrix (DBM), so named because the mineralized portion of the bone gets removed once it has been treated with an acid extraction in the sterilization process. This process leads to a decreased amount of growth factors limiting the osteogenic potential of allograft [41]. Thus, BMPs have been developed as a supplement to allografts to help with osteogenicity and create better fusion rates [42]. Allografts are rarely used by themselves, as data suggest that when combined with an autograft, better fusion rates occur [43-44]. Compared to an autograft, an allograft has an even higher risk of infection and rejection. Additionally, while BMPs have been postulated to have a synergistic effect with iliac crest bone grafts [45], there have been doubts regarding the cost-effectiveness of using them [46].

Present and future of bone grafts in spine fusion

Newer synthetic materials each have their own innate properties that make them valuable alternatives to bone grafts. Hydroxyapatite, coralline hydroxyapatite, and collagraft (Zimmer and Collagen Corporation) are all osteoconductive, but they are not osteogenic or osteoinductive [47]. Calcium phosphate and calcium sulfate cement are solely osteoinductive, which makes them good at filling metaphyseal holes, but they are expensive and unable to endure torsional and shear forces [48]. Autograft is the only option that has all three properties of being osteoconductive, osteoinductive, and osteogenic [49]. Table 1 lists various examples of bone substitutes and their properties.

There have been many recent developments of products and materials to be used as alternatives or additives to bone grafts. Bone graft substitutes, like demineralized bone matrix, are used instead of autografts. However, their potential to be osteoinductive, osteoconductive, and osteogenic is limited. Bone graft extenders are osteoconductive compounds used as additives to auto/allografts to increase bone graft volume and add structural support. Enhancers are compounds that have been developed to help with the fusion of bone grafts [41].

BMPs have been used as carriers for osteogenic growth factors. In 2009, there was a randomized controlled trial that compared the use of one BMP, called rhBMP-2, with iliac crest bone grafts to evaluate fusion rates in a posterior lumbar interbody fusion. The study showed that the rates of fusion were greater than that of the grafts used from the iliac crest [40]. Additionally, those who received iliac crest bone crafts tended to experience more blood loss and longer surgery times [50]. A potential risk of BMP use is paradoxical osteolysis, seroma formation, and recurrence of certain bone cancers.

While autografts remain the mainstay of bone grafting, synthetic bone substitutes and enhancers help combat some of the complications created by autografts. They help decrease donor site morbidity, risk of infection, and inflammation in the recipient tissue. The synthetic materials are constantly being adjusted for proper composition, making them more diverse [41].

The future of bone grafts will continue to develop. New materials are constantly being developed and evaluated. For example, Bhakta et al. took a new graft material made up of silane-modified polycaprolactone-tricalcium phosphate and heparin sulfate glycosaminoglycan, and they looked at the potential osteostimulatory properties in rats [51]. Likewise, Geurts et al. performed an in vitro study that showed support for autologous degenerative facet joint bone grafts to have very comparable osteogenicity to iliac crest bone grafts [52].

Today, patients benefit from a huge variety of bone graft techniques and options for spinal fusions. There are approximately 200,000 to 250,000 spinal fusion procedures in the United States annually [53-55]. Bone grafts are graded according to their ability to provide osteoinductivity, osteoconductivity, and osteogeneticity [25]. The surgeon has to choose according to the risk/benefit profile of patients and consider autograft, allograft, polymer-based, ceramic-based, growth factor-based, and cell-based bone grafts. The bone graft market is growing and is estimated to hit the $2 billion dollar mark in 2023 [56]. New options, such as mesenchymal stem cells (MSC), which can be found in different adult tissues and used as autografts for bony fusions, are gaining more popularity [57]. Nevertheless, despite these promising and interesting new bone graft options, ICBG remains the gold standard. 

## Conclusions

The field of bone grafts is evolving rapidly and is becoming increasingly complex. In the early days of bone grafting in spine surgery, bone grafts were used for tuberculosis patients and the structural support of the graft was the most important consideration. In light of modern research, bone grafts must meet specific criteria for successful implantation, and the spine surgeon must be aware of the risks and benefits of each option in order to provide the best choice for the patient.
